# Active Constituents of Psilocybin Mushroom Edibles

**DOI:** 10.1001/jamanetworkopen.2025.31345

**Published:** 2025-09-11

**Authors:** Richard B. van Breemen, Daniel Simchuk, Bjorn Fritzsche, Daniel Huson, Scott A. Kuzdzal, Jonathan Ferguson

**Affiliations:** 1 College of Pharmacy, Linus Pauling Institute, Oregon State University, Corvallis; 2 Rose City Laboratories, Portland, Oregon; 3 Shimadzu Scientific Instruments, Columbia, Maryland

## Abstract

This case series evaluates the active constituents of unregulated psilocybin mushroom edibles.

## Introduction

Psilocybin (4-phosphoryloxy-*N*,*N*-dimethyltryptamine) and its metabolite psilocin (4-hydroxy-*N*,*N*-dimethyltryptamine), hallucinogenic compounds produced by *Psilocybe spp* mushrooms, are under investigation for treating depression and substance use disorders.^1^ The use of psilocybin mushrooms is legal under regulated conditions in Oregon and Colorado, but to ensure safety, psilocybin-containing products require standardization and production using current Good Manufacturing Practices. However, unregulated products marketed as magic mushroom edibles (eg, gummies and chocolates) often contain no psilocybin and instead contain muscimol from *Amatina muscaria*, synthetic tryptamines, or other adulterants. During 2024, the Centers for Disease Control and Prevention reported 180 emergency cases, 73 hospitalizations, and 3 deaths associated with these products in 34 states.^2^ This study analyzed the constituents of unregulated magic mushroom commercial products.

## Methods

This case series included analysis of a select group of consumer products. Because no human participants were involved, this study was deemed exempt by the Oregon State University Institutional Review Board. Samples of 12 magic mushroom products (11 gummies and 1 chocolate) were purchased at retail shops in Portland, Oregon (Table 1). The gummies were extracted into acetonitrile using the dispersive solid phase extraction method QuEChERS, and the chocolate was extracted using methanol. Rose City Lab’s state of Oregon–accredited high performance liquid chromatography method was used to test for psilocybin and psilocin.^3^ Extracts were then analyzed using a Shimadzu gas chromatography–mass spectrometer, and peaks were tentatively identified using the National Institute of Standards and Technology/Environmental Protection Agency/National Institutes of Health (Electron Ionization) and Scientific Working Group for the Analysis of Seized Drugs mass spectral libraries. The structures of these compounds were confirmed, and additional compounds were identified using (1) ultra-high performance liquid chromatography (UHPLC) with high resolution mass spectrometry and (2) UHPLC with tandem mass spectrometry using 3 different mass spectrometers (Shimadzu 9030, 9050 quadrupole time-of-flight, and 8060 triple quadrupole). Results were then compared with standards (Table 2).

**Table 1.** Active Constituents of 12 Dietary Supplements Marketed as Psychedelic Mushrooms

Product	Active constituents disclosed on the product labels	Active constituents detected
Gummy 1	Muscarine, muscimol, nootropic proprietary mushroom blend	None
Gummy 2	None	Kavalactones: kavain, yangonin, dihydromethysticin, methysticin
Gummy 3	None	Caffeine
Gummy 4	Lion’s mane mushroom extract, reishi mushroom extract, cordyceps extract, proprietary microdosed nootropic blend	Cannabinoids: cannabidivarin, cannabidiol, cannabichromene, Δ^9^-tetrahydrocannabinol, cannabinol
Gummy 5	None	None
Gummy 6	None	Psilocin
Gummy 7	Psilocybin (100 mg per gummy)	None
Gummy 8	Mushroom blend extract	Synthetic psychoactive tryptamines: mipracetin, miproacetin isomer, 4-hydroxydiethyltryptamine, 4-hydroxydiethyltryptamine isomer
Gummy 9	None	Synthetic psychoactive tryptamines: mipracetin, mipracetin isomer, 4-hydroxydiethyltryptamine, 4-hydroxydiethyltryptamine isomer
Gummy 10	None	None
Gummy 11	Mushroom extract blend 250 mg, consisting of maitake, shiitake, lion’s main, reishi, cordyceps, chaga, turkey tails, white button, black fungus, royal sun mushroom	Psilocin
Chocolate 1	Lion’s mane mushroom, reishi mushroom	Psilocin

**Table 2.** Gas Chromatography–Mass Spectrometry (GC-MS) and High-Resolution Ultra-High Performance Liquid Chromatography With Tandem Mass Spectrometry Analysis of Active Constituents in Magic Mushroom Edibles

Product	Major constituents	GC-MS	LC-MS retention time, min	Measured monoisotopic mass *m/z*	Theoretical mass *m/z* [M+H]^+^	Elemental composition (ΔM ppm)
Gummy 1	None	NA	NA	NA	NA	NA
Gummy 2	Kavain	Yes	8.80	231.1034	231.1021	C_14_H_14_O_3_ (5.5)
	Dihydromethysticin	Yes	8.55	277.1070	277.1076	C_15_H_16_O_5_ (2.2)
	Yangonin	Yes	9.25	259.0989	259.0970	C_15_H_14_O_4_ (7.2)
	Methysticin	Yes	8.50	275.0933	275.0919	C_15_H_14_O_5_ (4.9)
Gummy 3	Caffeine	Yes	3.80	195.0891	195.0882	C_8_H_10_N_4_O_2_ (4.6)
Gummy 4	Cannabidivarin	Yes	12.20	287.2040	287.2011	C_19_H_26_O_2_ (10.0)
	Cannabidiol	Yes	13.25	315.2350	315.2324	C_21_H_30_O_2_ (8.2)
	Δ^9^-THC	Yes	13.46	315.2350	315.2324	C_21_H_30_O_2_ (8.2)
	Cannabinol	Yes	14.15	311.2012	311.2011	C_21_H_26_O_2_ (0.3)
	Cannabichromene	Yes	14.57	315.2354	315.2324	C_21_H_30_O_2_ (9.5)
Gummy 5	None	NA	NA	NA	NA	NA
Gummy 6	Psilocin	No	2.18	205.1344	205.1340	C_12_H_16_N_2_O (1.5)
Gummy 7	None	NA	NA	NA	NA	NA
Gummy 8	Mipracetin	No	4.4	275.1775	275.1759	C_16_H_22_N_2_O_2_ (5.6)
	Mipracetin isomer	No	4.9	275.1775	275.1759	C_16_H_22_N_2_O_2_ (5.6)
	4-Hydroxy-diethyl-tryptamine	Yes	2.9	233.1665	233.1653	C_14_H_20_N_2_O (4.8)
	4-Hydroxy-diethyl-tryptamine isomer	No	3.6	233.1665	233.1653	C_14_H_20_N_2_O (4.8)
Gummy 9	Mipracetin	No	4.4	275.1775	275.1759	C_16_H_22_N_2_O_2_ (5.6)
	Mipracetin isomer	No	4.9	275.1775	275.1759	C_16_H_22_N_2_O_2_ (5.6)
	4-Hydroxy-diethyl-tryptamine	Yes	2.9	233.1667	233.1653	C_14_H_20_N_2_O (5.6)
	4-Hydroxy-diethyl-tryptamine isomer	No	3.6	233.1667	233.1653	C_14_H_20_N_2_O (5.6)
Gummy 10	None	NA	NA	NA	NA	NA
Gummy 11	Psilocin	No	2.18	205.1344	205.1340	C_12_H_16_N_2_O (1.5)
Chocolate 1	Psilocin	No	2.18	205.1344	205.1340	C_12_H_16_N_2_O (1.5)

Abbreviations: LC-MS, liquid chromatography–mass spectrometry; ΔM ppm, mass error in parts per million; *m/z*, mass-to-charge ratio; [M+H]^+^, protonated molecule; NA, not applicable; THC, tetrahydrocannabinol.

## Results

Most of the product labels listed confectionary ingredients, but some listed active ingredients that were not detected or did not disclose any active ingredients (Table 1). The label of gummy 7 indicated psilocybin (100 mg per gummy), but none was detected in this or any of the products. The *Amanita muscaria* constituents muscarine and muscimol were listed on the label of gummy 1 but were not detected in any of the products.

We found 7 gummies and the chocolate were adulterated because they contained ingredients not disclosed on the labels (Table 1). Gummy 3 contained caffeine, whereas gummies 6 and 11 and the chocolate contained psilocin but not psilocybin or its biosynthetic precursors baeocystin and norbaeocystin, suggesting adulteration with synthetic psilocin. Based on comparison with an authenticated kava extract and identification of kavalactones (eg, kavain, dihydromethysticin, yangonin, and methysticin), gummy 2 contained an extract of kava (*Piper methysticum* Forst.). Kava is used for its anxiolytic and hallucinogenic properties but has been linked to hepatotoxicity.^4^ Gummy 4 contained an extract of *Cannabis sativa* L. based on the identification of cannabinoids, including cannabidiol, cannabidivarin, cannabichromene, Δ^9^-tetrahydrocannabinol, and cannabinol (Table 2).

Gummies 8 and 9 contained synthetic psychoactive tryptamines or syndelics that do not occur naturally in *Psilocybe spp*., including mipracetin (4-acetoxy-*N*-methyl-*N*-isopropyltryptamine), 4-hydroxy-diethyltryptamine, and isomers of each (Table 1 and Table 2). No active ingredients were detected in gummies 1, 5, 7, or 10.

## Discussion

Many unregulated retail magic mushroom edibles lack psilocybin and are adulterated with synthetic tryptamines, botanicals, or other undeclared compounds. The presence of syndelics—with unknown toxicology and pharmacology—raises significant safety concerns. Other adulterants also pose potential risks, especially when consumed unknowingly. Mislabeling and ingredient substitution endanger consumers and erode public trust in emerging psychedelic therapies. Given their widespread availability, including online distribution, there is urgent need for improved testing standards, stricter regulation, enhanced quality control, and state and federal enforcement.^2^ One study limitation is the small number of products tested. These findings are consistent with previous studies that found mislabeling and adulteration of *A. muscaria* and *Psilocibe spp*. products, including edibles.^5,6^

## Notes

Supplement.
Data Sharing Statement

## References

[1] Dinis-Oliveira RJ. Metabolism of psilocybin and psilocin: clinical and forensic toxicological relevance. Drug Metab Rev. 2017;49(1):84-91. doi:10.1080/03602532.2016.1278228 28074670

[2] Centers for Disease Control and Prevention. Environmental health studies: severe illness potentially associated with consuming Diamond ShruumzTM brand chocolate bars, cones, and gummies. November 4, 2024. Accessed July 8, 2025. https://www.cdc.gov/environmental-health-studies/outbreak-investigation-diamond-shruumz-products/index.html#cdc_generic_section_1-outbreak-investigation-summary

[3] Oregon Health Authority. Oregon Health Authority issues first psilocybin laboratory license. April 21, 2023. Accessed July 8, 2025. https://content.govdelivery.com/accounts/ORDHS/bulletins/3561877

[4] White CM. The pharmacology, pharmacokinetics, efficacy, and adverse events associated with kava. J Clin Pharmacol. 2018;58(11):1396-1405. doi:10.1002/jcph.1263 29791008

[5] Correia MS, Gonzaga MJ, Temple C, Gerona RR. Quantitative analysis of recreational psychoactive mushroom gummies in Portland, Oregon. Clin Toxicol (Phila). 2025;63(4):261-266. doi:10.1080/15563650.2025.2450240 39977248

[6] Katragunta K, Avula B, Chittiboyina AG, Lata H, Khan IA. Quantitative LC-QToF-MS analysis of mycochemicals in Amanita muscaria, Psilocybe spp. (Agaricomycetes), and consumer products. Int J Med Mushrooms. 2025;27(1):29-39. doi:10.1615/IntJMedMushrooms.2024056373 39717916

