# Supplementary material for: Active Constituents of Psilocybin Mushroom Edibles
Source: JAMA Netw Open. 2025 Sep 11;8(9):e2531345. doi: 10.1001/jamanetworkopen.2025.31345 (PMC12426793; doi:10.1001/jamanetworkopen.2025.31345)
Supplement: Supplement. — Data Sharing Statement [file jamanetwopen-e2531345-s001.pdf]

Data Sharing Statement

Data
Data available: Yes
Data types: Data (not involving human participants)
How to access data: vanbreer@oregonstate.edu
When available: With publication

Supporting Documents
Document types: None

Additional Information
Who can access the data: anyone requesting the data
Types of analyses: Mass spectra, mass chromatograms, and related analytical chemistry data
Mechanisms of data availability: with investigator support
